# The multi-kinase inhibitor CG-806 exerts anti-cancer activity against acute myeloid leukemia by co-targeting FLT3, BTK, and Aurora kinases

**DOI:** 10.21203/rs.3.rs-2570204/v1

**Published:** 2023-02-22

**Authors:** Guopan Yu, Weiguo Zhang, Hongying Zhang, Charlie Ly, Mahesh Basyal, William G. Rice, Michael Andreeff

**Affiliations:** The University of Texas MD Anderson Cancer Center; University of Texas MD Anderson Cancer Center; Aptose Biosciences; The University of Texas MD Anderson Cancer Center; The University of Texas MD Anderson Cancer Center; Aptose Biosciences; The University of Texas MD Anderson Cancer Center

**Keywords:** Multi-kinase inhibitor, acute myeloid leukemia, FLT3, CG-806

## Abstract

**Background:**

Despite the development of several FLT3 inhibitors that have improved outcomes in patients with FLT3-mutant acute myeloid leukemias (AML), drug resistance is frequently observed, which may be associated with the activation of additional pro-survival pathways such as those regulated by BTK, aurora kinases, and potentially others in addition to acquired tyrosine kinase domains (TKD) mutations of *FLT3* gene. *FLT3*may not always be a driver mutation.

**Objective:**

To evaluate the anti-leukemia efficacy of the novel multi-kinase inhibitor CG-806, which targets FLT3 and other kinases, in order to circumvent drug resistance and target *FLT3* wild-type (WT) cells.

**Methods:**

The anti-leukemia activity of CG-806 was investigated by measuring apoptosis induction and analyzing cell cycle with flow cytometry *in vitro*, and its anti-leukemia

**Results:**

CG-806 demonstrated superior anti-leukemia efficacy compared to commercially available FLT3 inhibitors, both *in vitro* and *in vivo*, regardless of *FLT3* mutational status. The mechanism of action of CG-806 may involve its broad inhibitory profile of FLT3, BTK, and aurora kinases. In*FLT3* mutant cells, CG-806 induced G1 phase blockage, while in *FLT3*WT cells, it resulted in G2/M arrest. Targeting FLT3 and Bcl-2 and/or Mcl-1 simultaneously resulted in a synergistic pro-apoptotic effect in *FLT3*mutant leukemia cells.

**Conclusion:**

The results of this study suggest that CG-806 is a promising multi-kinase inhibitor with anti-leukemia efficacy, regardless of *FLT3* mutational status. A phase 1 clinical trial of CG-806 for the treatment of AML has been initiated (NCT04477291).

## Introduction

Fms-like tyrosine kinase 3 (*FLT3*)-internal tandem duplication (ITD) mutations occur in approximately 30% of adult acute myeloid leukemia (AML) patients and are associated with poor clinical outcomes (1), particularly in patients with higher mutant-to-wild type (WT) allelic ratios (2). Several putative FLT3 inhibitors (FLT3is) such as sorafenib, midostaurin, quizartinib, crenolanib, and gilteritinib have been investigated alone and in combination with chemotherapeutic drugs or other targeted agents in pre-clinically and clinical trials (3, 4). However, as monotherapies, these inhibitors tend to have limited effectiveness and resistance to the drugs often develops quickly (4–9). The acquisition of secondary mutations in the tyrosine kinase domain (TKD) of *FLT3* is one mechanism of such resistance (6, 10), and has been identified in AML patients who relapsed or developed resistance to type-2 FLT3is, such as sorafenib and quizartinib (6, 11).

Moreover, aberrant activation of a number of other receptor and non-receptor tyrosine kinases has been linked to resistance to FLT3 inhibitors, including mitogen-activated protein kinases (MAPKs), signal transducer and activator of transcription 5 (STAT5), cellular myelocytomatosis oncogene (c-Myc) protein and Bruton’s tyrosine kinase (BTK) (12–15). Over-expression of c-Myc has been observed in most human hematopoietic malignancies and is associated with poor prognosis (16). The phosphorylation of c-Myc is regulated by the FLT3 downstream MAPK and PI3K/AKT signaling pathways (1). BTK is also part of the *FLT3*-ITD signalosome and is activated in an FLT3-ITD-dependent manner to induce proliferation in AML cells (17). In fact, targeting BTK with ibrutinib shows anti-proliferative effects in AML by mediating suppression of FLT3 downstream signaling MAPK, AKT, and NF-κB; and combined inhibition of FLT3 and BTK reportedly has additive anti-leukemia effects (17, 18).

Overexpression of aurora kinases (AURK), a family of serine/threonine kinases, has also been consistently demonstrated in a variety of leukemia cell lines and primary AML samples (19). The aberrant expression of AURK may be associated with poor-risk cytogenetic abnormalities and high blood cell counts in patients with AML (20). It has been reported that these CD34+/CD38− cells aberrantly expressed elevated levels of AURK that are required for cell cycle progression (21, 22), and targeting AURK demonstrated *in vitro* efficacy against human Myc-overexpressing AML cells (23). Concomitantly targeting AURK and FLT3 exhibited potent cytotoxicity with lower half-maximal inhibitory concentrations (IC_50_s) values against *FLT3*-ITD-mutant MV4–11, MOLM13 and MOLM13-resistant AML cells. The latter harbor ITD and D835Y dual mutations (24, 25). Interestingly, Druker’s group recently identified aurora kinase B as an early resistance factor to FLT3 inhibition (26). Therefore, co-targeting FLT3/AURK may be another potential therapeutic strategy in AML treatment. Furthermore, a therapeutic kinase inhibitor directed against all three novel targets, FLT3, BTK, and AURK, may be able to achieve much enhanced anti-leukemia efficacy in the targeted therapy of AML. Furthermore, FLT3 mutations may be acquired late in leukemogenesis and therefore may not be critical driver mutations. Activity against non-FLT3 mutant AML will then present a distinct advantage by targeting several subclones, FLT3 mutant and non-mutant.

The small molecule drug CG-806 (luxeptinib; Aptose, San Diego, CA) is a multi-kinase inhibitor with high activity against FLT3 mutations (27). Here, we evaluated the anti-leukemia activity of CG-806 by investigating its anti-proliferation and survival effects in AML *in vitro* as well as in AML xenograft models. We found that CG-806 has superior anti-leukemia effects against both *FLT3* WT and mutant AML, especially in AML harboring *FLT3* and TKD point mutations, which is a potential mechanism of secondary resistance to FLT3i. CG-806 profoundly inhibited phosphorylated (p)-BTK and p-AURK in addition to its FLT3 inhibition *in vitro* and significantly extended survival *in vivo* AML murine models. Our findings suggest that co-targeting FLT3, BTK, and AURK with a multi-kinase inhibitor CG-806 may be potent against AML regardless of *FLT3* mutational status.

## Methods

### Compounds

CG-806 was provided by Aptose Biosciences (San Diego, CA). Supplementary Fig. S1 shows the molecular structure of CG-806. Small molecule FLT3 inhibitors quizartinib, gilteritinib and crenolanib, AURK inhibitor SNS-314, Bcl-2 antagonist venetoclax and Mcl-1 inhibitor A1210477 were purchased from Selleckchem (Houston, TX).

### Leukemia cell lines and patient samples

AML cell lines were obtained from ATCC (Manassas, VA) or Deutsche Sammlung von Mikroorganismen und Zellkulturen (DSMZ; Braunschweig, Germany) and cultured according to instructions. The *FLT3* status of these AML cell lines is shown in Table 1. All the cell lines were validated by STR DNA fingerprinting using the AmpFISTR Identifier kit according to the manufacturer’s instructions (Applied Biosystems cat 4322288). Primary AML patient samples were obtained from The University of Texas MD Anderson Cancer Center under an IRB-approved protocol, with written informed consent and in accordance with the principles of the Declaration of Helsinki. Specific details are provided in the Supplemental Methods and Table S1.

### Cell viability and apoptosis assays

AML cell lines or primary AML samples were treated with indicated drugs for 48–72 h. The number of viable cells was determined with a Vi-CELL XR Cell Counter (Beckman Coulter Inc., Indianapolis, IN) using the Trypan blue dye exclusion method, and apoptosis induction was determined using flow cytometry by assessing annexin V positivity and propidium iodide (PI) positivity as described previously (28). The IC_50_s for cell growth inhibition and the half-maximal effective concentrations (EC_50_s) for apoptosis induction were calculated using CalcuSyn software (BioSoft, Cambridge, UK).

### Immunoblot analyses

Protein levels in treated cells were determined by immunoblot analysis as described previously (4). Briefly, treated cells were collected, cell lysates were prepared, resolved by electrophoresis on 10–12% precast sodium dodecyl sulfate-polyacrylamide gels, and transferred to Hybond-P membranes. After immunoblotting with antibodies, signals were detected using the Odyssey Infrared Imaging System (LI-COR Biosciences, Lincoln, NE) and semi-quantitative assessment was determined using the Scion Image system and software (beta version 4.03; Scion, Frederick, MD). Details in the Supplemental Methods.

## Cell cycle analysis

Cell cycle progression was determined using flow cytometric analysis of cellular DNA content and bromodeoxyuridine (BrdU) incorporation. Details in Supplemental Methods.

## c-Myc knockdown with siRNA transfection

The indicated siRNAs and mock control (scramble) siRNAs were purchased from Dharmacon Research, Inc. (Lafayette, CO). Transfections of MOLM14 leukemia cells were carried out by electroporation using the Amaxa Nucleofector system (solution V, program O-017; Lonza, Basel, Switzerland) following the manufacturer’s instructions. The final concentration of siRNA was 300 nM. After 24 h of transfection, the indicated concentrations of CG-806 were added to the cells for an additional 24 h of culture.

### Animal studies

An AML model was established by intravenously xenografting GFP-tagged Ba/F3-*FLT3*-ITD cells (0.5 × 10^6^ cells/mouse), infected with lentivirus expressing firefly luciferase, into NOD.Cg-Prkdcscid Il2rgtm1Wjl/SzJ mice (eight-week-old female) (The Jackson Laboratory, Bar Harbor, ME). The mice were treated with 10 mg/kg or 100 mg/kg CG-806 (oral gavage daily, 10 mice/group) starting on day 4 after leukemia cell injection, when an unambiguous luciferase signal was recorded. Control mice (n=10) received vehicle once daily for 5 weeks. Mice were noninvasively imaged using an Xenogen-200 *in vivo* bioluminescence imaging system (Xenogen, Hopkinton, MA) after being injected with 4 mg of luciferin substrate (D-luciferin, GoldBoi, St. Louis, MO). Bioluminescence images were obtained and quantitated as described previously (4). Three mice per group were humanely killed on day 11 after leukemia cell injection, and peripheral blood (PB) and bone marrow (BM) samples were collected to assess leukemia cell engraftment by measuring GFP positivity with flow cytometry. The *in vivo* studies were performed under approved animal care protocol and the standards of the Association for Assessment and Accreditation of Laboratory Animal Care.

### Statistical analyses

The Student *t*-test was used to analyze immunoblotting and cell apoptosis data. *P*-values ≤ 0.05 were considered statistically significant. All statistical tests were two-sided, and the results are expressed as the means of triplicate samples/experiments ± standard deviations or as means with 95% confidence intervals. Survival was estimated using the Kaplan–Meier method (29), and log-rank statistics was used to assess differences in survival between groups.

## Results

### CG-806 exhibits anti-leukemia activity superior to other FLT3i in samples with *FLT3* WT or TKD mutations through the inhibition of FLT3/AURK/BTK

**Figure 1:**
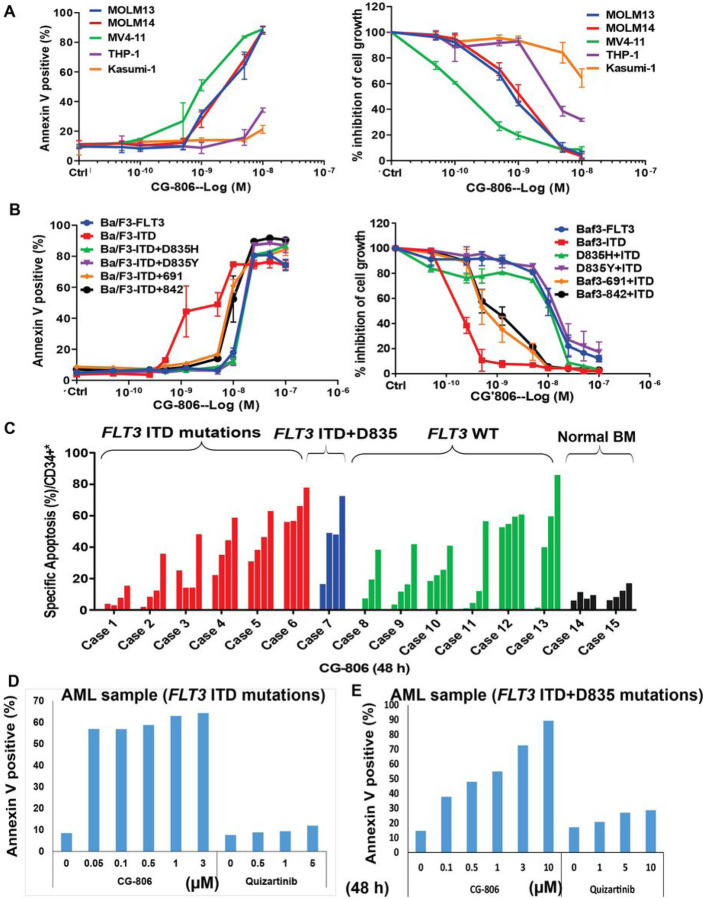
CG-806 demonstrates an anti-leukemia effect in both *FLT3* mutated and WT AML cell lines and primary samples. (A) Human and (B) murine leukemia cell lines with *FLT3*WT, *FLT3* ITD mutations, or dual *FLT3*-ITD + different TKD mutations were exposed to increasing doses of CG-806 for 72 h *in vitro*. Apoptosis induction and cell proliferation inhibition were assessed by measuring annexin V positivity with flow cytometry, and viable cells were assessed with a trypan blue exclusion assay. Experiments were performed in triplicate, and the results are presented as means ± standard deviations. (C) Primary AML mononuclear cell samples with different *FLT3* mutation statuses and normal BM samples were exposed to increasing doses (0, 0.1, 0.5, 1 and 3 μM) of CG-806 for 48 h. Apoptosis induction was assessed by measuring annexin V positivity in CD34+ cell population with flow cytometry. Data are presented as percentages of specific apoptosis induction (specific apoptosis (%) = 100 (drug-induced apoptosis - spontaneous apoptosis)/(100 - spontaneous apoptosis)) (57). Primary samples from an AML patient with *FLT3*-ITD mutations (D) and an AML patient with *FLT3*-ITD+D835 mutations (E) were treated with CG-806 or quizartinib *ex vivo* for 48 h, and apoptosis induction was measured with flow cytometry.

Our initial studies indicate that CG-806 has impressive kinase inhibition against WT and mutant FLT3, BTK, and AURK as well at extremely low nanomolar IC_50_s in a cell-free biochemical kinase inhibition assay (Table S2). Therefore, we first evaluated the anti-leukemia activity of CG-806 in AML cell lines with different *FLT3* mutation status. Treatment with extremely low doses (nanomolar to sub-nanomolar) of CG-806 for 72 h profoundly inhibited cell growth via apoptosis induction in both human and murine leukemia cell lines harboring either *FLT3*-ITD mutations or *FLT3*-ITD+TKD point mutations (Fig. 1A, B). Cells harboring *FLT3* TKD mutations or ITD + TKD dual mutations usually show resistance to most currently available FLT3i in previous studies (6, 10, 30). The IC_50_s and EC_50_s of CG-806 against these leukemia cell lines were in the low nanomolar to sub-nanomolar range (Table 1). Interestingly, whereas CG-806 had low IC_50_s (i.e., 4 to 10 nM) in most human and murine *FLT3*-WT leukemia cell lines, its EC_50_s could not be determined in some human *FLT3*-WT leukemia cell lines, such as THP-1 and Kasumi-1 (Table 1). Most importantly, CG-806 had profound pro-apoptotic effects in primary AML patient samples irrespective of *FLT3* mutation status, but did not induce apoptosis in BM cells from healthy donors (Fig. 1C). This suggested that CG-806 has broad and potent anti-cancer activity against AML cells in addition to a potential therapeutic window with respect to toxicity to normal cells.

To evaluate the anti-leukemia potency of CG-806, we compared the cytotoxic effects of the drug with other currently approved/available FLT3 and multi-kinase inhibitors in *FLT3*-mutated and *FLT3*-WT AML cell lines and patient samples. The IC_50_s of CG-806 were much lower than those of other FLT3is particularly in leukemia cells harboring the “gatekeeper” F691 mutation. The IC_50_s were 10.0 nM for CG-806 but 115.3 nM, 98.4 nM, and 257.6 nM for quizartinib, gilteritinib, and crenolenib, respectively (Table 2). We further compared the apoptogenic effect of CG-806 with that of the other FLT3i in AML patient samples *ex vivo*. CG-806 demonstrated markedly greater cytotoxicity than quizartinib in primary peripheral blood mononuclear cells with *FLT3*-ITD mutations or with *FLT3*-ITD+TKD mutations (Fig 1D, E).

**Figure 2:**
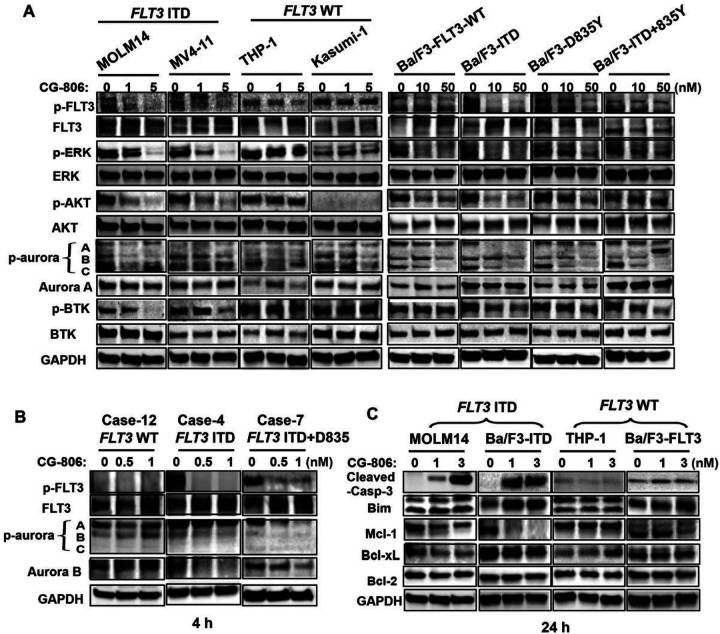
CG-806 suppresses p-FLT3, -BTK and –AuraK and triggers a pro-apoptotic effect in *FLT3*mutated AML cells. Leukemia cell lines (A) and primary AML cells (B) were treated with the indicated concentrations of CG-806 for 4 h. The expression levels of targeting-correlated proteins were measured using immunoblotting. (C) *FLT3*-ITD-mutated MOLM13 and Ba/F3 cells and *FLT3*-WT THP-1 and Ba/F3 cells were exposed to CG-806 for 24 h, and apoptosis-related proteins were measured with immunoblotting. GAPDH served as a loading control.

Immunoblot analyses demonstrated that CG-806 at nanomolar concentrations markedly suppressed phosphorylation levels of FLT3, AURK, and BTK, and their downstream signaling partners p-AKT and p-ERK in the leukemia cell lines and primary AML samples harboring *FLT3*-ITD mutations and/or *FLT3*-TKD mutations (Fig. 2A, B). CG-806 also upregulated the pro-apoptotic protein Bim in *FLT3* WT and ITD mutant AML cell lines after 24 h of treatment, and later triggered the cleavage of caspase-3 and PARP in *FLT3*-ITD-mutated AML cells (Fig. 2C, Supplementary Fig. S2). However, the treatment only marginally affected Bcl-2 and Bcl-xL levels in the leukemia cells, and even upregulated the anti-apoptotic protein Mcl-1 especially in Ba/F3-ITD mutant and Ba/F3-*FLT3*-WT cell lines (Fig. 2C).

### CG-806 blocks leukemia cells in G1 phase in *FLT3*-ITD-mutated AML cells and triggers G2/M arrest in *FLT3*-WT AML cells

**Figure 3:**
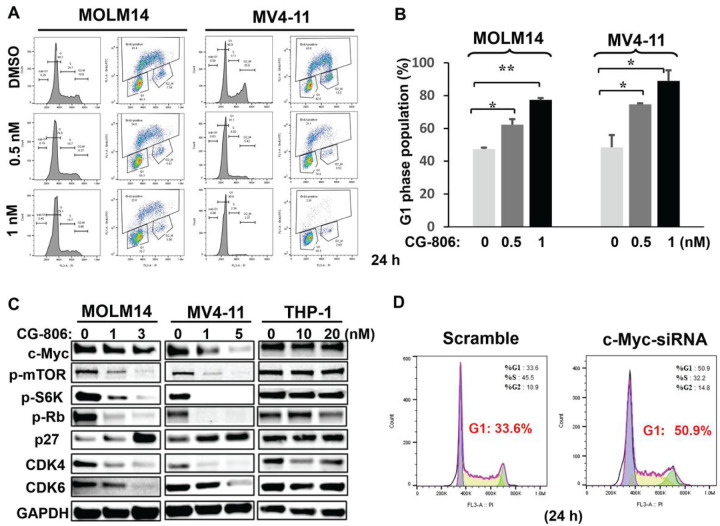
CG-806 blocks leukemia cells in G1 phase in *FLT3* mutant AML cells mainly through suppression of c-Myc. (A) Human *FLT3*-ITD-mutated MOLM14 and MV4–11 cells were treated with CG-806 for 24 h. The graphs show a representative figure of propidium iodide (PI)-stained and BrdU-labeled populations. (B) The G1 phase distribution was summarized from 3 independent experiments. Data are presented as the means ± standard deviations. *p ≤ 0.05, **p ≤ 0.01; Student *t*-test. (C) *FLT3*-ITD-mutated MOLM14 and MV4–11 cells and *FLT3*-WT THP-1 cells were treated with CG-806 for 24 h, and the indicated protein levels were measured with immunoblotting. (D) MOLM14 cells were treated with 300 nM c-Myc siRNA or with scrambled siRNA for 48 h, and the cell cycle was determined with flow cytometry after staining with PI.

To further characterize the mechanism(s) underlying the anti-leukemia activity of CG-806, we investigated the impact of CG-806 on cell cycle progression. Results indicated that CG-806 blocked cells in G1 phase in *FLT3*-ITD-mutated MOLM14 and MV4–11 leukemia cell lines after 24 h of treatment as determined by BrdU incorporation assay (Fig. 3A, B). Immunoblotting analyses showed profound suppression of cell proliferation-related proteins p-mTOR, -S6K, and -RB, upregulation of p27, and reduction of G1 phase checkpoint proteins CDK4, CDK6 and c-Myc as well (Fig. 3C). In terms of cell proliferation, c-Myc has key abilities to control cell cycle progression by promoting transcription of its downstream genes for cell cycle transition from G0/G1 into S phase and antagonizing cell cycle inhibitor activity (31). Therefore, we further determined if c-Myc is critical for CG-806-induced G1 arrest. Knocking down c-Myc with siRNA in MOLM14 cells (Supplementary Fig. S3) triggered more pronounced G1 phase arrest compared to MOLM14 cells without c-Myc knockdown (50.9% vs. 33. 6% in MOLM14-cMyc-siRNA vs. MOLM14-cMyc-scramble cells, respectively, *p* < 0.05) (Fig. 3D), implying that c-Myc suppression has a role in CG-806-induced inhibition of cell growth through G1 phase arrest in *FLT3*-mutated leukemia cells.

**Figure 4:**
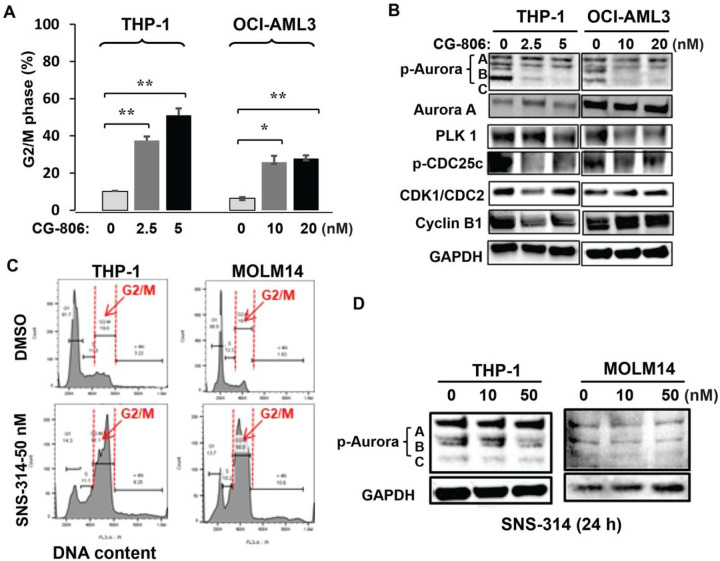
CG-806 induces G2/M arrest through p-ARUK inhibition in *FLT3*-WT AML cells. (A) *FLT3*-WT THP-1 and OCI-AML3 cells were exposed to the indicated concentrations of CG-806 for 24 h, and PI staining was used to measure the cell cycle (DNA content) distribution. Data are presented as the means ± standard deviations of 3 independent experiments. *p ≤ 0.05, **p ≤ 0.01; Student *t*-test. (B) *FLT3*-WT leukemia cells were treated with CG-806 for 24 h, and cell cycle-related proteins were determined with immunoblotting. *FLT3*-WT THP-1 cells and *FLT3*-ITD-mutated MOLM14 cells were treated with the aurora inhibitor SNS-314 (10 or 50 nM) or with DMSO for 24 h. DNA content and cell cycle distribution were determined by flow cytometry after PI staining (C), and p-aurora protein levels were assessed by immunoblotting (D).

However, we did not observe G1 arrest in *FLT3*-WT cells. Conversely, CG-806 inhibited the growth of *FLT3*-WT THP-1 and OCI/AML3 cells by triggering significant G2/M arrest instead (Fig. 4A). Immunoblotting analyses demonstrated that CG-806 profoundly suppressed p-AURK B and C levels and downregulated Polo-like kinase 1 (PLK1), p-CDC25c, and cyclin B1 (Fig. 4B). To confirm that AURK inhibition was associated with G2/M arrest in *FLT3*-WT cells, we suppressed AURK activity by using an AURK specific inhibitor SNS-314 (32) in either *FLT3*-WT or -mutant AML cell lines THP-1 or MOLM14. Results showed a similar G2/M arrest accompanied by p-AURK inhibition in both cell lines (Fig. 4C, D), which suggests that AURK suppression and G2/M arrest are interconnected regardless of *FLT3* mutation status in AML cells.

### CG-806 has marked anti-leukemia efficacy in murine models of *FLT3*-mutated leukemia

**Figure 5:**
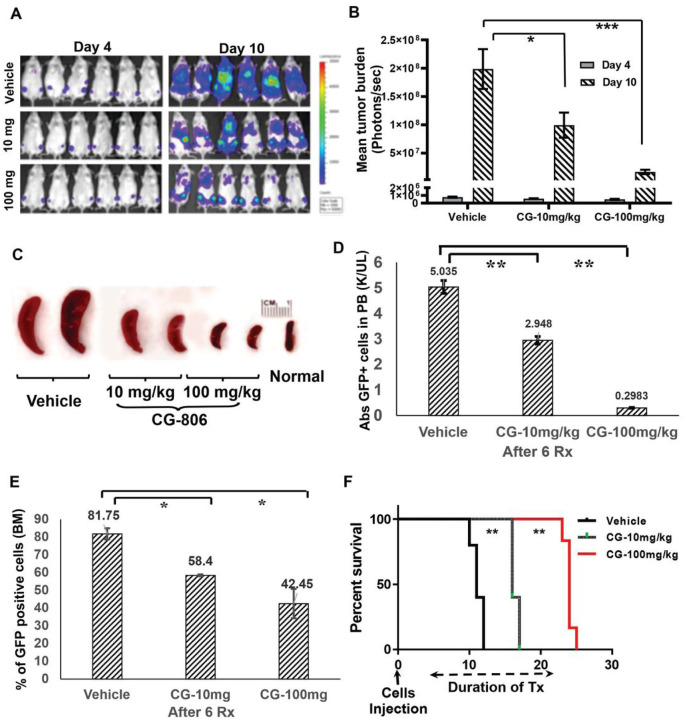
CG-806 exerts profound anti-leukemia effects in a mice model xenografted with *FLT3*-ITD-mutated leukemia cells. (A) Serial bioluminescence images of representative mice treated with CG-806 (10 or 100 mg/kg) or vehicle acquired 4 and 10 d after leukemia cell injection are shown. (B) Quantitative analysis of leukemia burden based on the data of serial bioluminescence images. (C) Spleen size from different treatment groups of mice was compared after 1 wk of CG-806 treatment. “Normal” means normal mice without leukemia cells injection and drug treatment. The engraftment of GFP+ leukemia cells was evaluated as the absolute number of GFP+ cells in PB (D) and the percentage of GFP+ leukemia cells in bone marrow (E), as assessed with flow cytometry. (F) The Kaplan-Meier method was used to assess the median survival of each group, and log-rank statistics were used to assess differences in survival. *p ≤0.05, **p ≤ 0.01, ***p ≤ 0.001; Student *t*-test.

Our preliminary data indicated that mice receiving 100 mg/kg CG-806 had high plasma concentrations 24 h after one dose (Supplementary Fig. S4). Therefore, we established a leukemia model in NSG mice by xenografting Baf3-*FLT3*-ITD cells and treated mice with 10 or 100 mg/kg doses of CG-806. CG-806 significantly reduced the leukemia burden by 48% (10 mg/kg dose; *p* < 0.05) and 93% (100 mg/kg dose; *p* < 0.001) compared to the vehicle group (Fig. 5A, B) and eliminated leukemia-related splenomegaly after 1 week of drug administration (Fig. 5C). CG-806 also eliminated leukemic blasts in both PB and BM in a dose-dependent manner (Fig. 5D, E). In addition, the survival duration of the 10 mg/kg (16 d) and 100 mg/kg CG-806 groups (24 d) was significantly longer than that observed in the vehicle group (11 d; p < 0.01) (Fig. 5F). CG-806 at either dose did not affect mouse body weight (data not shown).

### Bcl-2 and/or Mcl-1 inhibition profoundly enhances the cytotoxic effects of CG-806 in AML cells

**Figure 6:**
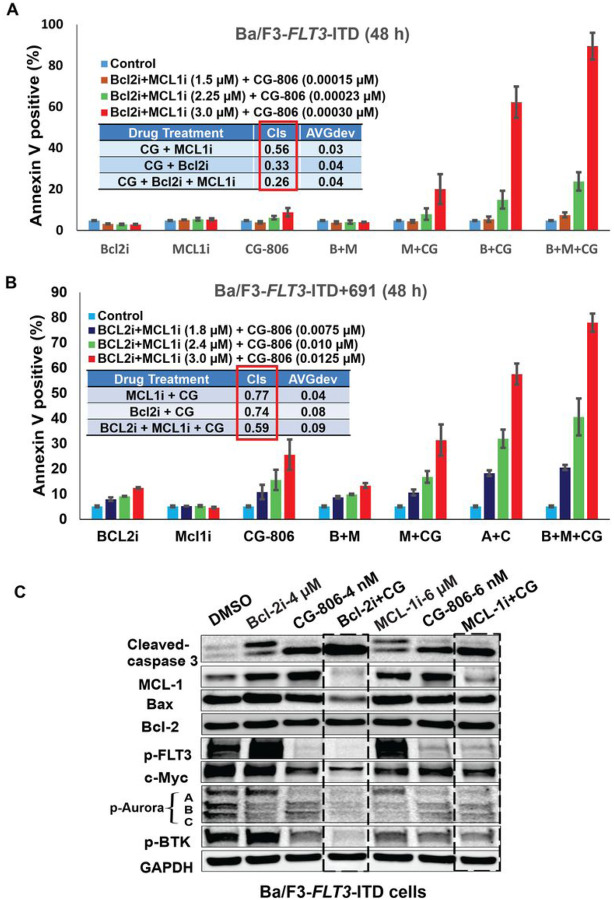
Bcl-2 and Mcl-1 inhibitors enhance the anti-leukemia effects of CG-806. *FLT3*-ITD-mutated Ba/F3 leukemia cells (A) and *FLT3*-ITD+F691-mutated Ba/F3 leukemia cells (B) were exposed to CG-806 and Bcl-2 antagonist venetoclax and/or Mcl-1 inhibitor A1210477 for 48 h. Cell apoptosis induction was assessed with flow cytometry by measuring annexin V positivity. Data are from three independent experiments and presented as the means ± standard deviations. (C) Immunoblotting was used to assess the targeting-correlated proteins in *FLT3*-ITD-mutated Ba/F3 cells after being exposed to the combination regimens for 24 h. Bcl-2i (B) = Bcl-2 inhibitor; Mcl-1i (M) = Mcl-1 inhibitor; CG = CG-806.

CG-806 demonstrated much more pronounced anti-proliferative than pro-apoptotic effects, accompanied by only marginal inhibition of Bcl-2, Bcl-xL, and Mcl-1 as well in most of the tested leukemia cell lines, even upregulated the anti-apoptotic protein Mcl-1 in Ba/F3-*FLT3*-WT and ITD mutant cells (Fig. 2C). In fact, the overexpression of Mcl-1, Bcl-2 and Bcl-2A1 has been associated with therapy resistance of AML cells (33, 34). This finding provides a rationale for combining CG-806 with Mcl-1 and/or Bcl-2 inhibitors to improve anti-leukemia efficacy as previously shown for other FLT3i (35). Therefore, we sought to determine whether the pro-apoptotic effect of CG-806 could be enhanced by combination with Bcl-2 and/or Mcl-1 inhibitors. As expected, all combinations of CG-806 with the Bcl-2 inhibitor venetoclax and/or the Mcl-1 inhibitor A1210477 showed markedly synergistic pro-apoptotic effects in leukemia cells; the combination indexes (CIs) in Ba/F3-ITD, Ba/F3-ITD+F691L and Ba/F3-*FLT3*-WT cell lines were 0.33 ± 0.04, 0.74 ± 0.08, and 0.36 ± 0.10, respectively, for the combination with Bcl-2 inhibitor; 0.56 ± 0.03, 0.77 ± 0.04, and 0.73 ± 0.07, respectively, for the combination with Mcl-1 inhibitor; and 0.26 ± 0.04, 0.59 ± 0.09, and 0.42 ± 0.04, respectively, for the three-drug combination (Fig. 6A, B, Supplementary Fig. S5).

Immunoblot analyses showed that targeting Bcl-2 and/or Mcl-1 concomitantly with CG-806 profoundly suppressed Mcl-1, reduced p-FLT3, -BTK, and -AURK, and triggered a marked cleavage of caspase-3 (Fig. 6C), suggesting that the combination regimens trigger potent leukemia cell killing which may translate in beneficial effect in relapsed/refractory AML regardless of *FLT3* mutational status.

## Discussion

Several FLT3i have been developed over the last two decades including sorafenib (4, 5) and FDA-approved gilteritinib and midostaurin (8, 36). A newer generation FLT3i, crenolanib is still under development and showed impressive anti-leukemia effects against resistant AML harboring *FLT3* TKD mutations (7). One complicating factor is the oligoclonal nature of AML (37, 38). *FLT3* mutant clones co-exist with *FLT3* WT clones or dysregulated signaling, which creates a much more complex scenario. Meanwhile, it also provides a window into therapeutic vulnerabilities. Aberrant expression of survival signaling pathways such as MAPK, BTK and AURK is associated with resistance to FLT3i (12, 15, 19). CG-806 as a novel multi-kinase inhibitor of targeting FLT3/BTK/AURK might provide a better pharmacological notion for overcoming resistance than that more specific FLT3i. In the present study, we demonstrated that CG-806 has a superior anti-leukemia efficacy especially against AML harboring “gatekeeper” F691 mutations or *FLT3* WT compared to other FLT3i, without detectable toxicity in normal BM samples. Mechanistically, CG-806 profoundly suppresses FLT3, BTK, and AURK activation simultaneously and results in impressive cytotoxicity in these AML cells, particularly against *FLT3*-WT AML cells which demonstrated abnormally high expression of aurora kinase. Our findings confirmed that CG-806 is a pan-FLT3 inhibitor that may benefit from the simultaneous suppression of FLT3, BTK and AURK activation, leading to a promising anti-leukemia effect against AML regardless of their *FLT3* status.

Further, we found that CG-806 achieved its anti-leukemia activity in *FLT3*-mutated AML and *FLT3*-WT AML through different mechanisms. Specifically, CG-806 induced G1 arrest in *FLT3*-mutated MOLM14 and MV4–11 cells but induced G2/M arrest in *FLT3*-WT THP-1 cells at IC_50_s of about 1 nM and 5 nM, respectively. In *FLT3*-mutated cells, G1 cell cycle progression is closely associated with the dominant activation of FLT3 and its downstream effectors, AKT/mTOR/S6K, and MAPK. These signaling cascade proteins are highly expressed and constitutively activated in *FLT3*-mutated leukemia cells. In addition, BTK, which is expressed in about 80% of human AML, mediates FLT3-ITD-dependent Myc and STAT5 activation (17), and transcriptionally increases the levels of G1 cell cycle checkpoint proteins through c-Myc signaling (39). Thus, by co-targeting FLT3 and BTK, which are dominantly activated in *FLT3*-mutated AML, CG-806 enhances the downregulation of c-Myc to trigger G1 arrest. In fact, Eriksson *et al*. reported that targeting FLT3 signaling with the FLT3i AKN-028 or midostaurin for 12–48 h triggered G1 phase arrest in *FLT3*-mutated MV4–11 AML cells as well (40). In the present study, targeting FLT3 with quizartinib or gilteritinib or targeting BTK with ibrutinib also triggered G1 arrest in *FLT3*-ITD-mutated AML cells (data not shown). Similarly, c-Myc knockdown elicited G1 arrest in *FLT3*-mutated MOLM14 cells as well. These findings strongly imply that the CG-806-induced suppression of FLT3 and BTK and their downstream signaling, and the subsequent downregulation of c-Myc play critical roles in G1 arrest in *FLT3*-mutated AML cells.

We did not observe c-Myc repression in *FLT3*-WT AML cells treated with CG-806 although it has a high expression in the *FLT3*-WT cells. In fact, *FLT3*-WT cells had higher expression of p-AURK, but lower p-FLT3 or -BTK compared to *FLT3*-mutated AML cells (Supplementary Fig. S6). An investigation of genes essential for proliferation and survival of cancer cells with CERES (computational method to estimate gene-dependency levels from CRISPR–Cas9 essentiality screens) dependency score (lower score indicates a higher likelihood that the gene of interest is essential in a given cell line) indicated that *FLT3*-WT leukemia cell lines have low CERES scores of AURK-B (−2.0 and −1.7, respectively, on Kasumi-1 and THP-1 cell lines) and of AURK-A (−1.45 and −1.37, respectively, in Kasumi-1 and OCI/AML3 cell lines); which suggests a higher dependency on AURK with regard to proliferation and survival of *FLT3*-WT leukemia cell lines (41). Actually, the overexpression of AURK and their downstream PLKs were reported to be associated with tumorigenesis of many human tumors, including leukemias (21, 42). AURK signaling plays a fundamental role in regulating cell cycle checkpoints that ensure the timing and order of cell cycle events such as DNA repair, bipolar spindle formation, chromosome segregation and mitotic exit (43–45). Therapeutic targeting of AURK and PLK1 prevents the completion of mitosis and results in G2/M phase arrest and apoptosis (46). In the present study, AURK inhibition by CG-806 and the suppression of PLK1 in *FLT3*-WT cells THP-1 and OCI-AML3 were accompanied by a reduction in p-CDC25C and the subsequent inactivation of CDC25C, resulting in G2/M arrest. In fact, Jagtap *et al*. reported that suppressing AURK-B with dual AURK-B/FLT3 inhibitor (Compound-54) can trigger G2/M arrest in *FLT3*-ITD-mutated MOLM13 cells as well (24). In addition, the suppression of AURK activation by a specific AURK inhibitor SNS-314 (32) also triggered G2/M arrest in both *FLT3*-WT and mutant leukemia cells, which further supports the notion that AURK suppression is associated with G2/M arrest in leukemia cells. Similarly, in another study, the multi-kinase inhibitor midostaurin triggered G1 arrest in *FLT3*-mutated leukemia cells and G2/M arrest in *FLT3*-WT leukemia cells, although the mechanisms involved remain unresolved (47). It has been reported that midostaurin triggers G2/M arrest in breast cancer cell lines through inhibition of the AURK family proteins (48), which supports the notion of an association of AURK suppression and G2/M arrest in *FLT3*-WT AML. Actually, CG-806 demonstrates much higher anti-AURK activity than midostaurin (IC_50_s are 3 nM vs. 300 nM, respectively in CG-806 *vs*. midostaurin) (49), suggesting a more potent against AML with *FLT3*-WT.

In line with our *in vitro* results, CG-806 had robust anti-leukemia activity in a murine xenograft model of leukemia created using the aggressive *FLT3*-ITD-mutated Ba/F3 cells (Fig. 5), and in a PDX AML model as well (50). Compared with vehicle, 100 mg/kg CG-806 significantly reduced leukemia burden and the absolute leukemia cell count in PB by up to 17-fold after 1 wk of treatment. CG-806 also conveyed a remarkable survival benefit, as the survival duration of mice that received 10 mg/kg or 100 mg/kg CG-806 (16 and 24 d, respectively) were markedly longer than that of mice that received vehicle (11 d). Impressively, even at doses of up to 450 mg/kg, the daily oral administration of CG-806 for 14 d elicited no obvious toxicity in Balb/c mice (data not shown). These results suggest that CG-806 could be well-tolerated in a clinical setting as monotherapy or as a combinatorial drug with other targeted agents or chemotherapeutics for AML treatment.

We further observed that combinations of CG-806 with the Bcl-2 antagonist venetoclax and/or the Mcl-1 inhibitor A1210477 had synergistic pro-apoptotic effects not only in *FLT3*-mutated AML cells (both those with *FLT3*-ITD mutations and those with *FLT3*-ITD + “gatekeeper” F691 mutations), but also in *FLT3*-WT cells, indicating that the combinatorial regimens may have a promising anti-leukemia efficacy in both *FLT3*-WT and mutant AML. In fact, combinations of venetoclax with various other drugs, including FLT3i, are being actively investigated in AML (35, 51, 52). The anti-apoptotic protein Mcl-1 is one of the main determinants of venetoclax resistance in AML (53). The downregulation of Mcl-1 sensitizes AML to Bcl-2 inhibitor-induced leukemia cell killing (54), and we have also reported a highly synergistic effect of venetoclax with Mcl-1 inhibition recently (33). In the present study, CG-806 by itself showed modest Mcl-1 inhibition, indicating a need for pharmacological inhibition of Mcl-1 which was indeed observed. Also, the benefit was observed in the synergistic efficacy of combination with venetoclax and/or A1210477, through activation of the intrinsic apoptosis pathway, evidenced by the increased level of cleavage caspase-3 cleavage.

Approximately 36 % of patients treated with gilteritinib develop resistance related to the emergence of *RAS* mutations (55). Ras mutations are also an established resistance factor for Bcl-2 inhibition (56). It remains to be seen if CG-806 can suppress the outgrowth of *RAS*-mutant subclones in AML, alone or in combination with venetoclax.

Taken together, CG-806 demonstrates superior anti-leukemia activity *in vitro* and *in vivo* compared to other FLT3i by suppression of FLT3/BTK/AURK simultaneously. Mechanistically, CG-806 triggers G1 phase arrest in FLT3-mutated AML cells by predominantly targeting FLT3 and BTK, and G2/M phase arrest in FLT3-WT AML cells by predominantly targeting AURK. CG-806 exerts robust anti-leukemia activity by efficiently reducing leukemia burden and doubling survival in a murine xenograft model. Targeting FLT3 mutant and -wild-type cells is a potential additional advantage. Furthermore, our findings provide a potential strategy for CG-806 in combination with Bcl-2 and/or Mcl-1 inhibitors for AML therapy. Currently, CG-806 is in phase 1 clinical trial (NCT04477291) for patients with relapsed or refractory AML and higher-risk myelodysplastic/myeloproliferative neoplasms as well.

## Figures and Tables

**Table 1.** Activities of CG-806 Against *FLT3* mutated and *FLT3* WT Acute Leukemia Cells

	Cell Line	Characteristics	IC_50_, nM	95% Lower/Upper	EC_50_, nM	95% Lower/Upper
Murine Cells	Ba/F3-FLT3	*FLT3* WT	9.49	6.04/14.9	23.22	15.66/34.43
	Ba/F3-ITD	*FLT3* ITD	0.30	0.07/1.29	5.60	2.31/13.58
	Ba/F3-D835G	*FLT3* D835G	0.12	0.02/0.89	4.30	2.20/8.41
	Ba/F3-D835Y	*FLT3* D835Y	8.26	4.18/16.30	15.46	8.22/29.09
	Ba/F3-ITD+691	ITD+F691L	0.43	0.31/0.61	14.65	8.84/24.28
	Ba/F3-ITD+842	ITD+Y842C	0.73	0.42/1.27	13.39	9.24/19.42
	Ba/F3-ITD+D835Y	*FLT3-*ITD+D835Y	9.72	5.46/17.30	22.01	9.51/50.95
	Ba/F3-ITD+D835H	*FLT3-*ITD+D835H	6.74	3.71/12.26	25.82	14.25/46.78
Human Cells	MOLM13	*FLT3* ITD, t(9:11)	0.82	0.79/0.87	4.34	1.93/9.86
	MOLM14	*FLT3* ITD, t(9:11)	0.92	0.76/1.11	3.90	2.61/5.84
	MV4–11	*FLT3* ITD, t(4:11)	0.17	0.12/0.25	1.69	1.30/2.20
	OCI/AML3	*FLT3* WT	11.81	6.33/22.01	77.24	31.84/187.32
	THP-1	*FLT3* WT, t(9:11)	3.88	2.16/6.98	NA*	NA
	Kasumi-1	*FLT3* WT	21.99	16.38/29.53	NA	NA

* NA = not reachable

**Table 2.** IC_50_s comparison of CG-806 and other FLT3 inhibitors in *FLT3* WT and mutated cells.

FLT3 inhibitors	IC_50_ in Transfected Ba/F3 Cells (nM, n = 3)				
	*FLT3* WT^a^	*FLT3* ITD	*FLT3* D835Y	*FLT3*ITD+D835Y	*FL73* ITD+F691L
**CG-806**	**11.3**	**0.5**	**8.8**	**19.3**	**10**
**Quizartinib**	1956.0	2.2	2089	246.4	115.3
**Gilteritinib**	500.3	26.5	472.5	6.8	98.4
**Crenolanib**	2617.0	35	888.9	31.7	257.6

a *FLT3* WT cells are IL3-dependent and presented during the inhibitor treatment.

## Data Availability

The data that support the findings of this study are available from the corresponding author, [M.A.] upon reasonable request.

## Key Points

1. Multi-kinase inhibitor CG-806 exerts superior anti-leukemia activity in AML regardless of its *FLT3* status.
2. CG-806 triggers G1 arrest in *FLT3* mutated cells and G2/M arrest in *FLT3* WT cells through suppression of FLT3/BTK and aurora kinases.
3. Concomitantly targeting FLT3 and Bcl-2 and/or Mcl-1 exerts synergistic pro-apoptotic effects on both *FLT3* WT and mutated AML cells.
